# Shipping blood to a central laboratory in multicenter clinical trials: effect of ambient temperature on specimen temperature, and effects of temperature on mononuclear cell yield, viability and immunologic function

**DOI:** 10.1186/1479-5876-9-26

**Published:** 2011-03-08

**Authors:** Walter C Olson, Mark E Smolkin, Erin M Farris, Robyn J Fink, Andrea R Czarkowski, Jonathan H Fink, Kimberly A Chianese-Bullock, Craig L Slingluff

**Affiliations:** 1 Human Immune Therapy Center, University of Virginia, Charlottesville, VA, USA; 2 Dept. of Public Health Sciences, University of Virginia, Charlottesville, VA, USA; 3 Atlantic Research Group, 125 S. Augusta Street, Suite 3000, Staunton, VA, USA; 4 1901 E. Market Street, Charlottesville, VA, USA; 5 9652 S. Michigan, Chicago, IL, USA; 6 JVI, LLC, 615 Cami Lane, Charlottesville, VA, USA; 7 Dept. of Surgery, University of Virginia, Charlottesville, VA, USA

## Abstract

**Background:**

Clinical trials of immunologic therapies provide opportunities to study the cellular and molecular effects of those therapies and may permit identification of biomarkers of response. When the trials are performed at multiple centers, transport and storage of clinical specimens become important variables that may affect lymphocyte viability and function in blood and tissue specimens. The effect of temperature during storage and shipment of peripheral blood on subsequent processing, recovery, and function of lymphocytes is understudied and represents the focus of this study.

**Methods:**

Peripheral blood samples (n = 285) from patients enrolled in 2 clinical trials of a melanoma vaccine were shipped from clinical centers 250 or 1100 miles to a central laboratory at the sponsoring institution. The yield of peripheral blood mononuclear cells (PBMC) collected before and after cryostorage was correlated with temperatures encountered during shipment. Also, to simulate shipping of whole blood, heparinized blood from healthy donors was collected and stored at 15°C, 22°C, 30°C, or 40°C, for varied intervals before isolation of PBMC. Specimen integrity was assessed by measures of yield, recovery, viability, and function of isolated lymphocytes. Several packaging systems were also evaluated during simulated shipping for the ability to maintain the internal temperature in adverse temperatures over time.

**Results:**

Blood specimen containers experienced temperatures during shipment ranging from -1 to 35°C. Exposure to temperatures above room temperature (22°C) resulted in greater yields of PBMC. Reduced cell recovery following cryo-preservation as well as decreased viability and immune function were observed in specimens exposed to 15°C or 40°C for greater than 8 hours when compared to storage at 22°C. There was a trend toward improved preservation of blood specimen integrity stored at 30°C prior to processing for all time points tested. Internal temperatures of blood shipping containers were maintained longer in an acceptable range when warm packs were included.

**Conclusions:**

Blood packages shipped overnight by commercial carrier may encounter extreme seasonal temperatures. Therefore, considerations in the design of shipping containers should include protecting against extreme ambient temperature deviations and maintaining specimen temperature above 22°C or preferably near 30°C.

## Background

Cell-based immunological assays are integral to monitoring the effects of immunotherapy clinical trials. The main clinical specimen obtained for these assays is whole blood collected in heparinized vacutainer tubes from which peripheral blood mononuclear cells (PBMC) are isolated. Assays of cellular immune responses to immune therapy depend on functional and viable PBMC. It is critical that outside factors, other than study parameters, do not introduce significant variability in the immune assays due to compromised PBMC integrity. Therefore, trials utilizing multiple clinical centers present challenges in how to best process and transport whole blood and tissue samples.

The need for specific guidelines for the shipment of biological specimens is of great concern for the conduct of multi-center clinical trails at the national and international level [1-3]. Both complex processing and delay before processing by individual laboratories increase the variability in specimen performance [4]. In contrast, central laboratory processing lessens the variability introduced by multiple processing protocols but is more costly and may not be available for all investigators. It therefore becomes a critical issue in the design of multi-center clinical trials to determine whether biological specimens should be processed immediately, the same day, or after shipment to a central laboratory.

Early studies have demonstrated how time and temperature of storage affect lymphocyte viability and phenotype when whole blood is stored overnight at 4°C [5-7]. Storage at room temperature prior to processing also affects viability and blastogenic responses [8] as well as lymphocyte separation by Ficoll density centrifugation [9,10]. The importance of establishing standard shipping parameters has been stressed in the infectious disease setting, in which a profound impact of shipping was noted on the lymphoproliferative responses to microbial antigens in both HIV-infected and healthy donors [11,12]. Single cell-based techniques such as ELIspot assays [13-15], intracellular cytokine staining [16-19], and HLA-specific multimeric assays [20-22] are widely used and depend on optimal conditions for specimen handling in order to detect rare populations of peptide specific lymphocytes in response to immunotherapy. Several studies have confirmed that cryopreserved PBMC can be used reliably in these assays [23-26]. Use of cryopreserved samples, however, depends on optimal sample handling before and after cryopreservation. Some studies have defined optimal time intervals between venipuncture and cryopreservation [26-29] and optimal conditions for freezing [30]. Also, handling and storage of cryopreserved PBMC have been evaluated, showing that fluctuations in sub-zero freezing temperatures can alter the viability and function of recovered lymphocytes; shipping conditions for frozen samples have also been addressed [31,32]. However, the effect of ambient temperature changes during shipping or storage prior to cryopreservation has not been addressed.

It has been suggested that an interval of whole blood storage exceeding 8 hours (h) causes a significant decrease in cellular immune function [27]. This finding provides rationale for immediate isolation and cryopreservation of PBMC at each participating clinical center and indeed, optimization of cryopreservation media and of thawing practices has improved recovery of immunological responses at the single cell level [25,30]. However, processing of blood and cryopreservation of PBMC at off-site locations is expensive and requires oversight and quality control of the processing lab at each center. Thus, for many multicenter clinical trials of cancer vaccines and other therapies, all off-site whole blood specimens are shipped to a central laboratory according to a standard operating protocol, and monitored strictly for quality control and quality assurance. Our concern that shipping whole blood in different seasons, in various climates, may impact PBMC viability and function prompted this study. Specifically, we have addressed the effect of shipping temperatures on cell viability, recovery and function, and have modeled these in vitro when controlling for temperature.

## Methods

### Blood collection, processing and storage

Patients' blood specimens were derived from participants enrolled in one of three studies. Participants were enrolled in the clinical studies following informed consent, and with Institutional Review Board for Health Sciences Research approval (IRB-HSR# 10598, 10524, and11491) and review by the FDA (BB-IND# 9847 and 12191). Patients' blood specimens from 2 clinical trials (HSR# 1524(HSR# 10524 and 11491) were monitored during a 9 month period from late summer, through fall, winter and early spring. Two hundred and eighty-five blood specimens collected at participating clinical trial centers in Houston, TX and Philadelphia PA, were shipped to Charlottesville VA. Clinical laboratory analyses, including complete blood counts (CBC) and differential hematological counts, were performed at the individual centers and the results incorporated into a trial database. An additional 60 ml of blood were collected in 10cc heparinized vacutainer tubes (BDBiosciences, Franklin Lakes, NJ) and were shipped, in insulated packaging, by overnight courier at ambient temperature to the Biorepository and Tissue Research Facility (BTRF) at the University of Virginia (UVa) for processing and cryo-preservation, on the day they arrived, for future immunological testing in cell-based assays. Shipments of patients' blood specimens were continuously monitored using the TempCheck Sensor (Marathon Products, Inc., San Leandro, CA) to determine the temperature range to which blood samples were exposed when shipped overnight by commercial carrier, and to evaluate the effects of those temperatures on cell yield. Temperature gauges recorded the maximum and minimum temperatures attained inside the packages during shipment. Blood drawn at UVa was processed either the same day or the following day, depending on when in the day it was drawn. The volume of blood collected, and the number of viable PBMC isolated were recorded by the BTRF. These values were used to determine the cell yield before cryo-preservation. In all cases, the PBMC fraction of whole blood was collected from Leucosep™ (Greiner Bio-One, Monroe, NC) tubes following centrifugation for 10 minutes at 1000 × g.

The expected cell yield for each sample was calculated from the CBC and differential tests performed on whole blood at the originating clinical laboratory. The absolute lymphocyte and absolute monocyte counts calculated from the CBC and differential were combined and multiplied by the volume of blood collected to represent the expected total PBMC in the blood (expected cell yield). Additional File 1 provides a table of cell count data from each center. The table shows the calculated percentage (mean, median, and quartiles) of lymphocytes and monocytes derived from differential and complete cell counts. The number of PBMC isolated by Ficoll separation, divided by the expected cell yield provides the ratio cell yield. Ratio cell yields of less than 1 are expected due to losses in Ficoll separation. However, because the Ficoll separations were done by the same central laboratory and according to a consistent protocol, differences in ratio cell yields in different subgroups of specimens are primarily attributed to effects of shipping conditions.

### Incubation conditions for whole blood

In one set of experiments, approximately 7-8 ml whole blood were collected into each of eleven heparinized vacutainer tubes from six healthy donors according to IRB protocol 10598 and were labelled to define the temperature conditions to which they would be exposed. Each tube was incubated at various temperatures over a 24 h period at conditions intended to model what may happen in overnight shipping conditions (Additional File 2). After a 1-2 h equilibration period at room temperature (RT, 22°C), tubes from each sample were placed in each of the 4 conditions: (a) temperature-controlled refrigerated centrifuge set at 15°C, (b) 22°C as a control condition, (c) water bath set at 30°C, or (d) water bath set at 40°C. In addition, one tube was placed in a 50°C water bath for 2 h, but this condition invariably led to hemolysis and the samples were not evaluable. For each temperature condition (other than RT), one tube was exposed to that low or high temperature for 2, 8 or 12 h, and then each was returned to RT for the remaining 24 h study period. Thus, one tube served as an untreated control and was at kept at RT for the whole 24 h. After these incubations, PBMC were isolated from each blood sample by Ficoll density gradient as described above. Viable cell numbers were determined by trypan dye exclusion. PBMC were cryopreseved in freezing medium (90% FCS, 10% DMSO) overnight at -80°C, then transferred to vapor phase liquid nitrogen for 1-4 weeks before thawing for analysis.

### ELIspot Assay

Cells producing IFNγ after antigen specific and non-specific stimulation were enumerated by ELIspot assay as described previously [33,34]. In brief, PBMC were thawed in pre-warmed RPMI1640 (Invitrogen, Carlsbad CA) containing 10% human AB serum (HuAB; Gemini) and 100 Units/mL of DNase I (Worthington Biochemical Corp., Lakewood, NJ). Cells were centrifuged at 350 × g and adjusted to the desired cell density in RPMI 1640 supplemented with 10% HuAB serum and plated into PVDF-membrane plates coated with anti-interferon gamma antibody (Pierce-Endogen, Thermo Scientific, Rockford IL). Phytohemagglutinin (PHA), phorbol myristate acetate (PMA and ionomycin were obtained from Sigma-Aldrich (St. Louis, MO). A pool of 35 MHC Class I restricted peptides consisting of peptides from cytomegalovirus, Epstein-Barr and influenza virus proteins (CEF peptide pool; [35]; Anaspec, Fremont CA) or media alone were added in quadruplicate and cultures incubated overnight at 37°C in a 5% CO_2 _atmosphere. Spots were developed according to standard protocol and enumerated on a BioReader 4000 (Bio-Sys, Karben, Germany) plate reader.

### Flow cytometry

CD3, CD4, CD8 and CD56 positive lymphocyte populations were enumerated by flow cytometry using fluorescent-labelled antibodies (BDBiosciences, San Diego, CA). Cells were washed, suspended in PBS (Invitrogen) containing 0.1% BSA (Sigma) and 0.1% sodium azide (Sigma). Titrated amounts of each reagent were added to cells, incubated, washed free of excess stain, and fixed in paraformaldehyde. To determine whether there was an increase in apoptosis due to different storage conditions, thawed PBMC were incubated overnight at 37°C in 5% CO_2 _in RPMI 1640 + 10% Human AB serum_. _The next day, PBMC were surface stained with fluorescently labeled antibodies to CD3, CD4, and CD8, then stained with Annexin V according to manufacturer's instructions (BDBioscience, San Diego, CA) and 7-AAD (EMD Chemicals, Inc., Gibbstown, NJ) to determine the level of apoptosis [36-38]. Cells were acquired on a FACSCalibur flow cytometer maintained by the Flow Cytometry core facility of the University of Virginia. Data were analyzed with FlowJo software (Treestar, Ashland OR).

### Testing of Blood Shipping Packages

The standard shipping container used in our clinical trials was obtained from Safeguard Technologies Corp. (Conshohocken, PA). It consisted of a white corrugated box fitted with a hydrophilic foam-lined clear plastic snap-lock case inserted into a plastic zip lock bag. This was placed inside a cardboard shipping container lined with 1" thick Styrofoam. An alternate packaging design was provided by JVI (Charlottesville VA) and consisted of a 14" × 11" × 5" box of 200# corrugated cardboard insulated with Control Temp Packaging foam of 1" thickness. Inside was placed a 12" × 9" × 3" clamshell type clear plastic box containing an 11" × 8.5" × 5/8" foam vial holder.

Each type of shipping container was tested for its ability to maintain temperature in cold ambient conditions (e.g.: during winter months). Forty heparinized vacutainers were filled with water and equilibrated to 37°C. Ten vacutainers were placed inside each of 4 packages (2 of each type). Each package type received a gel pack conditioned at either 37°C or 22°C which was then placed alongside the vacutainer holder. One probe of an indoor/outdoor thermometer (Taylor Precision Products, Oak Brook IL) was placed inside the package while another remained outside to monitor external ambient temperature. Packages were placed either in a cold room at 4°C for a minimum of 12 h or were handled in a manner to model the experience of a package being shipped via motor vehicle overnight in a non-heated compartment. Temperatures were recorded every 15 minutes during the first hour, and 30-60 minutes thereafter.

Additional testing of the JVI packaging material was performed by R.N.C. Industries Inc. (Norcross GA 30071) at high external package temperature. The clamshell foam holder containing vials of liquid was placed inside the package. Two 12 oz Control Temp gel packs conditioned at 20°C were placed in the clamshell onto which the foam vial holder (including the 1/4" foam above and below) containing five 5/8" vials filled with water conditioned at 20°C was placed inside. The package was closed, put at 45°C and the internal package temperature was monitored for 48 hours using an Omega OMB-DAQ-55 USB data acquisition system, serial number #156772. T thermocouples were calibrated 2 months earlier using a stirred water bath calibration.

### Statistical analysis

The MIXED procedure in SAS 9.1.3 (SAS Institute, Cary, NC) was used to analyze the effects of temperature (3 levels) and duration (3 levels) on outcomes including ELIspot, phenotype, and viability. These effects were modeled jointly (main effects plus interactions) for each outcome measure and outcome measurements were first normalized by division of the raw data by the donor value at RT for 24 h. Since donors served as blocks and contributed an observation from each condition (i.e. each combination of temperature and duration level), intra-donor correlation was modeled assuming a compound symmetry structure in the residual covariance matrix. Degrees of freedom were calculated using the Kenward-Roger method. To assess the effects of storage under different temperature conditions on apoptosis among CD4 and CD8 populations, a modeling scheme similar to the one above was performed using calculated logits as the outcome measure. This is defined as the log_e_([p_i_/1-p_i_]/[p_c_/1-p_c_]) where p = the proportion of cells that are apoptotic or necrotic (as defined by Annexin V and 7AAD staining); i = the storage conditions of the whole blood specimen; and c = the storage condition of the control specimen at RT for 24 hours. All tests were assessed at α = 0.05.

## Results

### Effect of shipping temperatures and extreme changes in temperature on the cell yield for clinical trial specimens

**Figure 1:**
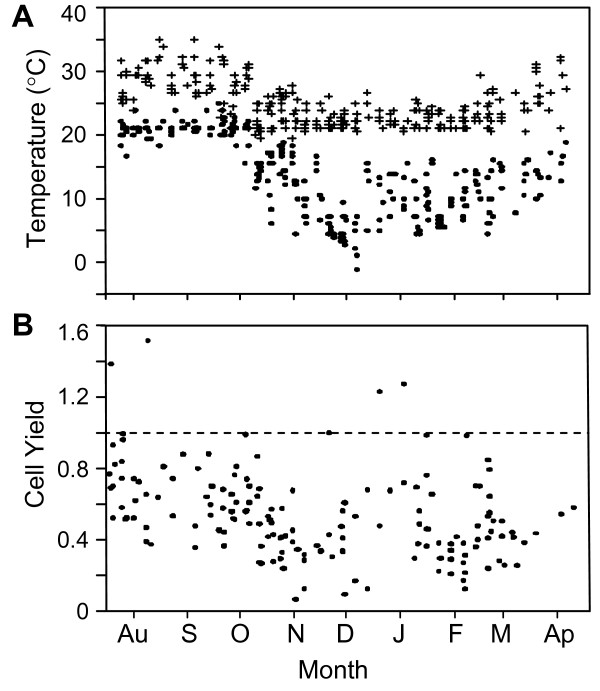
**Recorded internal package temperatures during shipment and cell yields of blood from off-site cancer centers**. (A) High (+) and low (●) package temperatures recorded between August, 2005 through April, 2006. (B) Yield of PBMC (cell yield) obtained from specimens shipped during this time after Ficoll separation. The ratio cell yield is expressed as a ratio of total number of PBMC collected after Ficoll divided by the number of PBMC (lymphocytes and monocytes) estimated from the differential WBC recorded on the same specimens before shipment. The dashed line represents 100% recovery of PBMC after Ficoll as a ratio cell yield of one.

Package temperatures were lowest in winter months and highest in summer months, suggesting that the temperatures experienced during shipping varied by ambient seasonal temperatures (Figure 1A). The extreme temperatures ranged from about -1°C to 35°C with 91% falling completely within the range of 4°C and 32°C.

**Figure 2:**
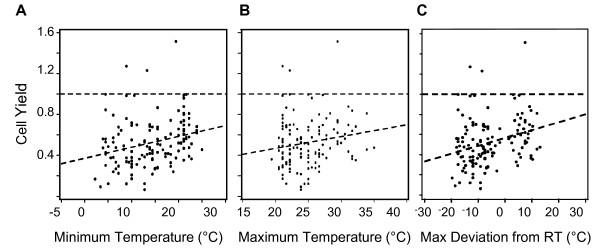
**The recovery of cells after Ficoll separation increased as shipping temperature increased**. (A) Correlation of the ratio cell yield with minimum temperature during transport; p = 0.001. (B) Correlation of the ratio cell yield with maximum temperature during transport; p = 0.04. (C) Correlation of the ratio cell yield as a function of maximum temperature deviation from room temperature (22°C) during shipment; p < 0.001

There was a trend to lower PBMC yields in colder months from November through February (Figure 1B), although outliers were noted. Lower minimum temperature was associated with lower cell yield (p = 0.001, Figure 2A), whereas higher maximum temperature correlated with higher cell yield (p = 0.04, Figure 2B). The range in shipping temperatures during the winter was typically bounded by a high temperature of 22°C, and during the warmer months by 22°C as a low temperature. The maximum change (deviation) in temperature from 22°C observed during shipment was determined using the high or low temperature furthest from 22°C. This represents an estimate of the degree of temperature fluctuation encountered during shipment and is plotted against the yield in Figure 2C, where there was a correlation with warmer temperatures (p < 0.001). Overall, warmer temperatures favored greater cell yields. These observations led us to initiate controlled in vitro studies on the impact of storage temperature on cell recovery, viability, and immunological function.

### Effect of temperature on cell yield before cryopreservation

To determine whether exposure to extreme temperatures impacts the overall integrity of PBMC, blood specimens from 6 normal volunteers were stored at temperatures in a range encountered during blood shipment or varying lengths of time and were assessed for cell yield, cell recovery and cell function (Additional File 2). Blood was exposed to temperatures of 15°, 30°, 40°, or 50°C for 2 h, 8 h, and 12 h and left at room temperature after that exposure for a total of 24 h after collection. Significant and unacceptable lysis and cell loss were associated with incubation 2 h at 50°C; thus, these were not analyzed further (unpublished observation). Adequate data already exist for the negative effects of refrigeration at 2-8°C [6,7,9]; so this temperature was not assessed here. Blood stored 24 h at room temperature (22°C) was used as a reference for comparison. A significant decrease in the PBMC cell yield was observed for samples stored at 15°C for 12 h (p < 0.003; Table 1). Blood stored at 30°C had PBMC yields almost identical to the RT standard. Exposure to high or low temperature for 8 h, followed by RT incubation was associated with no significant decrement in cell yields at any of the temperatures. There was a trend to lower cell yields with 12 h at 40°C, but it was not significant.

**Table 1:** Effect of exposure to different incubation conditions on PBMC isolation from whole blood and recovery after cryo-preservation

	Cell Yield before Cryopreservation			Cell Recovery after Thawing		
**Exptl** **RT**	**2 h** **22 h**	**8 h** **16 h**	**12 h** **12 h**	**2 h** **22 h**	**8 h** **16 h**	**12 h** **12 h**

15°C	0.85 (0.60, 1.10) p = 0.22	0.88 (0.63, 1.13) p = 0.32	0.59 (0.34, 0.84) **p = 0.003**	1.00 (0.70, 1.30) p = 0.99	1.02 (0.72, 1.32) p = 0.88	0.66 (0.36, 0.97) **p = 0.031**

30°C	0.90 (0.65, 1.15) p = 0.40	1.02 (0.76, 1.27) p = 0.90	1.00 (0.75, 1.25) p = 1	1.12 (0.82, 1.42) p = 0.41	1.20 (0.90, 1.50) p = 0.19	1.19 (0.88, 1.49) p = 0.21

40°C	0.87 (0.62, 1.12) p = 0.30	0.83 (0.58, 1.08) p = 0.16	0.79 (0.53, 1.06) p = 0.12	0.91 (0.61, 1.22) p = 0.56	0.78 (0.48, 1.08) p = 0.14	0.63 (0.32, 0.95) **p = 0.026**

Blood from 6 normal donors was incubated 24 h at RT (22°C, control), and for 2-12 h at 15, 22, 30 or 40°C (Exptl = Experimental), then at RT for the remainder of 24 h. PBMC were harvested and, cryopreserved, and thawed at least 1 week later. The estimated means and 95% confidence intervals of ratios of cell yield to the control sample (RT × 24 h) are shown, both before cryopreservation and upon recovery of cells after cryopreservation. P-values are in boldface when statistically significant.

### Effect of Temperature on Cell Recovery after cryopreservation

We hypothesized that shipping temperatures may impact cell recovery and viability after storage in liquid nitrogen. The total number of viable cells (trypan blue dye exclusion) was recorded for each of the PBMC samples exposed to varied temperatures as reported above. Percent recovery was calculated as the ratio of recovered viable cells to the number of viable cells initially frozen. Each condition was compared to storage at RT for 24 h. Significant reduction of PBMC recovery was associated with storage of blood 12 h at 15°C or 40°C but not with either 2 h or 8 h (Table 1). However, at 30°C, the trend favored higher recoveries of PBMC, at all time points, than that seen at RT.

### Effect of temperature on viability and phenotype after cryo-storage

These samples were also assessed by flow cytometry for evaluable PBMC populations and the selective loss of T lymphocyte sub-populations after cryo-preservation. Changes in the PBMC population were not reflected in the proportion of CD4^+ ^and CD8^+ ^lymphocyte sub-populations (Additional file 3) or in the proportion of CD56^+ ^lymphocytes (data not shown) compared to that seen when whole blood is stored overnight at RT.

**Figure 3:**
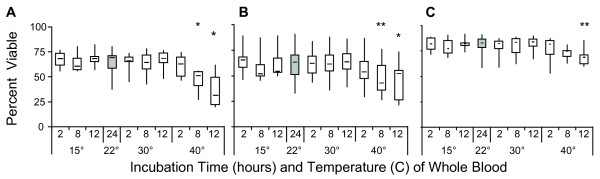
**Viability of PBMC 24 hours after thawing from liquid nitrogen**. After whole blood was incubated at different temperatures for varying lengths of time, PBMC were isolated and cryopreserved. Samples were thawed and rested overnight at 37°C before staining with CD4, CD8, Annexin V and 7-AAD. The viable populations were defined as Annexin V negative and 7AAD negative and are expressed as a percentage of the respective populations of (A) PBMC, (B) CD8 and (C) CD4 lymphocytes. Shaded area on graph represents the control condition of incubating whole blood at 22°C for 24 hours to which all other conditions were compared. (*) p = 0.003; (**) p = 0.03.

**Figure 4:**
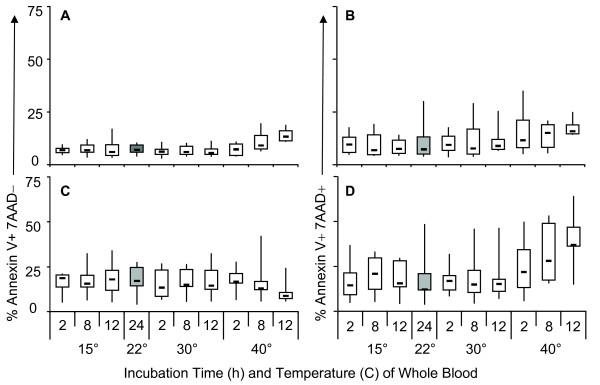
**CD8 T cells show greater susceptibility to apoptosis than CD4 T cells**. The percentage of cells in different stages of apoptosis was evaluated for CD4 and CD8 T cell populations. (A) Percentage of CD4 lymphocytes in early stages of apoptosis (Annexin V+, 7AAD-) and (B) late stages of apoptosis (Annexin V+, 7AAD+); (C) CD8 lymphocytes in early stages of apoptosis (Annexin V+, 7AAD-) and (D) late stages of apoptosis (Annexin V+, 7AAD+). Shaded region indicates control condition as described in Figure 3.

However, damage to cells as a result of extreme shipping temperatures may not be evident at the time of collection or immediately after cryo-storage, but rather during subsequent incubation [39]. Therefore, PBMC were assessed for viability using Annexin and 7AAD to measure apoptosis [36-38] after an overnight rest. Significant decreases in viable PBMC (Figure 3A) were observed in blood specimens incubated at 40°C for 8 h (p = 0.002) and 12 h (p < 0.001). This was not seen at the other temperature conditions tested, even at 12 h of incubation. CD8 populations (Figure 3B) showed significant decreases in viability at 40°C for 8 h (p = 0.013) and after 12 h (p = 0.03). CD4 viability (Figure 3C) was significantly reduced after 12 h at 40°C (p = 0.03). A greater proportion of CD4 T cells (Figure 4A and 4B) were in early stages of apoptosis (Annexin V+, 7AAD-) whereas a greater proportion of CD8 T cells (Figure 4C and 4D) were in the later stages of apoptosis (Annexin V+, 7AAD+) under these same conditions. Estimates of the odds ratio for CD4 and CD8 populations to undergo apoptotic or necrotic cell death after exposure to 40°C at 8 and 12 hours, had significance levels of p = 0.0335 and p = 0.0035 for CD4 populations, and p < 0.006 for CD8 when compared to the control storage condition (24 h @ RT).

### Effect of temperature on cell function after cryostorage

**Figure 5:**
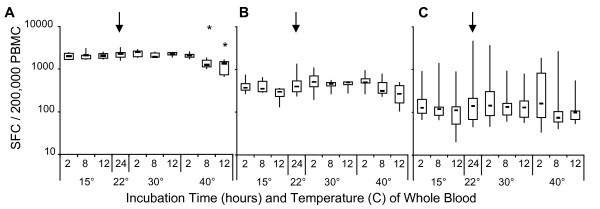
**Mitogen and antigen-activated PBMC responses as detected by IFNgamma secretion in an ELIspot assay**. After thawing from liquid nitrogen, PBMC were incubated 18 hours at 37°C with (A) PMA/ionomycin, (B) PHA or (C) CEF peptide pool and then tested for IFNg secretion by ELIspot assay. Results are presented as SFC per 200,000 PBMC for PMA and PHA. CEF SFC are adjusted for the percentage of CD8+ T cells and presented as SFC per 200,000 CD8 T cells. Each condition is compared to the control condition (arrows) as described in Figure 3. (*) p < 0.004.

The principal cell based assay for monitoring our clinical trials of immunotherapy is the ELIspot assay which measures specific T cell responses by enumerating T cells secreting cytokine (IFN-gamma) after peptide stimulation. We determined whether there was an adverse effect of temperature on the function of lymphocytes in our standard ELIspot procedure. Thawed PBMC from each temperature condition were stimulated overnight with PMA, PHA, or CEF or were left un-stimulated. The following day, plates were developed and the number of spots recorded for each condition. Relative to blood incubated overnight at RT, whole blood initially incubated at 40°C for 8 h and 12 h resulted in significant decreases in the number of IFN-gamma producing T cells in response to PMA (Figure 5A; p≤004). Lower spot counts to PHA (Figure 5B) and to CEF (Figure 5C) were observed with whole blood exposed to either 40°C or 15°C, respectively, for 12 h, but were not statistically significant. Incubation at 30°C for up to 12 h was equivalent to 22°C for measures of function by ELIspot.

### Package testing in high and low ambient temperatures

**Figure 6:**
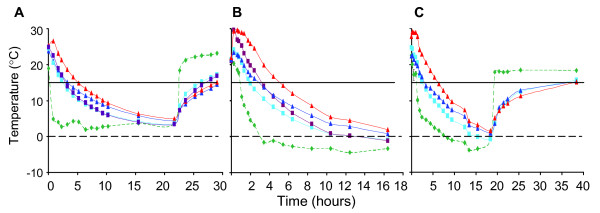
**Internal temperature change over time in containers designed for shipping blood specimens**. Ten water-filled vacutainer vials were pre-warmed to 37°C placed inside the JVI Control Temp shipping container or in the Safeguard (SG) shipping container, surrounded with pre-warmed gel packs, placed inside an insulated corrugated cardboard container, and sealed with tape for testing at low external temperatures. Internal package temperatures were continuously monitored inside JVI and SG shipping containers while placed (A) at a constant low temperature of 4°C for 22 hours followed by 22°C for 8 hours; (B) outdoors in ambient winter temperatures for 16 hours; and (C) outdoors in ambient winter temperatures for 18 hours followed by placement of package at 22°C for 20 hours. (green diamond) External package (ambient) temperature; internal package temperatures: (red triangle), JVI with 37°C thermal pack; (purple square), SG with 37°C thermal pack; (blue triangle), JVI with 22°C thermal pack; (blue square), SG with 22°C thermal pack. Solid black line indicates 15°C; dashed line denotes 0°C.

Packaging was designed by JVI (Charlottesville, VA) for shipping blood specimens in vacutainer tubes where high or low ambient temperatures may be encountered during shipping. Testing in our laboratory compared the internal temperatures in shipping containers designed by JVI with that of our prior shipping container (SafeGuard) under winter temperature conditions. Three of the four tests are presented in Figure 6. Pre-warmed gel packs (RT or 37C) were included to delay a rapid decrease in the internal temperature. Each shipping container was fitted with internal and external temperature probes and placed at 4°C or outside. In each condition, the internal temperatures in both types of containers fell at approximately the same rate (Figure 6A-C, representing 3 of 4 experiments that were performed). The JVI shipping container, compared to the SafeGuard container, maintained internal temperatures above 15°C more consistently. Gel packs conditioned at 37°C maintained an internal temperature above 15°C for approximately 2 hours longer than RT-conditioned gel packs when packages were placed at a constant external temperature of 4°C (Table 2). When exposed to outside temperatures as would occur during shipment in winter, gel packs pre-warmed at 37°C helped maintain an internal temperature above 15°C for 1.8 hours longer than gel packs conditioned at room temperature. Thereafter, the decline of the internal temperature was similar in all packaging conditions tested. After moving the packages to RT, the rates at which the internal temperatures increased were similar for each condition (Figure 6A, C).

**Table 2:** Pre-warmed gel packs extend the time above 15°C when shipping at cold temperatures

Outside Temperature	Gel Pack Temperature	Hours	
		
		Safe-Guard	JVI
4°C	37°C	3.5	4.5
	
	RT	1.8	2.0

Ambient	37°C	3.4	5.9
	
	RT	2.5	3.2

Gel packs pre-warmed at RT or 37°C were packed inside blood specimen shipping containers along with probes to measure the internal and temperature after sitting overnight in a constant 4°C cold room or outdoors where temperatures fell below freezing. The number of hours the internal temperature remained above 15°C is show.

**Figure 7:**
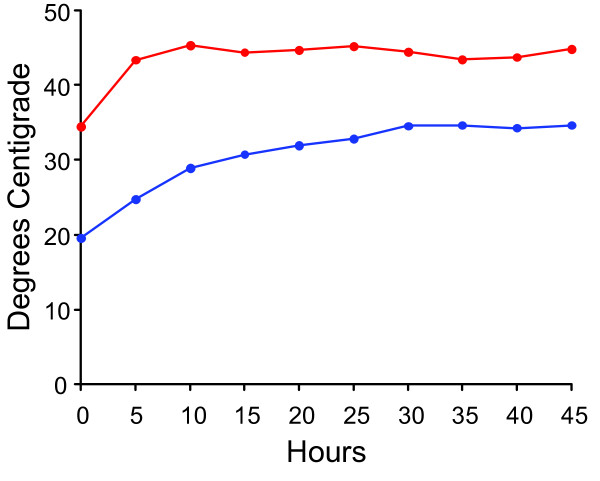
**Temperature performance test of the JVI Control Temp shipping container**. Five vials, filled with water conditioned at 20°C, were suspended inside the foam vial holder and placed inside the plastic clamshell plastic box fitted with small foam pads. Two of the vials each had a T thermocouple taped to it. The clamshell package was put inside the insulated corrugated cardboard box in which two 12 oz. Control Temp gel packs conditioned at 20°C were also placed inside and taped shut. The shipping container was set inside a 45°C chamber for forty-five hours and the internal package temperature recorded as described in Methods. The red line indicates the external temperature of the chamber. The blue line represents the average internal temperature of the shipping container obtained from duplicate temperature probes.

The effects of extreme high ambient temperatures on maintaining internal temperatures within the range of 15-35°C was tested on the newly designed JVI shipping container. The shipping container was placed at 45°C (Figure 7) and for 45 hours, the temperature remained under 35°C. For at least 21 hours, the internal temperature stayed between 20° and 30°C.

## Discussion

Recently much-needed attention has been given to the conditions under which blood specimens, collected for correlative studies of immune therapy, are handled prior to PBMC isolation. How samples are processed and shipped from trial sites as whole blood or separated PBMC can affect the outcome of immunological monitoring of vaccine-based immunotherapeutic clinical trials. Arguably, it is optimal to assay a blood sample immediately and at the site where it is collected, as is done for most routine clinical laboratory tests. However, for novel or experimental correlative studies, this is not usually feasible, since expertise for those tests requires specialized laboratories. Also, an argument can be made for evaluating pre- and post-treatment blood samples in the same assay to provide internal controls. Thus, blood samples often are shipped to centralized laboratories for correlative studies where they are often cryopreserved for later batch analysis. Another question is whether cryopreservation should be done at each site, or whether whole blood should be shipped to the central lab for processing there. Several details of cryopreservation methods can impact PBMC function and viability [30]; so if cell isolation and cryopreservation is done at each site, there needs to be intensive training and quality assurance to confirm comparable methods and results. Though it is an option, this approach often is infeasible for financial and organizational reasons. Thus, it is common for whole blood to be shipped from multiple sites to a central laboratory for PBMC isolation and cryopreservation, for later analysis. However, the possible impact of temperature during shipping, and prior to processing, has not been systematically addressed. In this study, we have focused on the effect of temperature during shipping to assess its variation based on season of the year, and to assess the impact of temperature on PBMC viability and function.

In multiple studies in the HIV literature, delayed processing of whole blood has been identified as a major factor affecting PBMC performance in cell-based immunological assays [26-29]. Delay in processing during overnight shipping (at least 24 h) decreased responses to microbial antigens in lymphoproliferative assays [12] indicating the need for defined transportation conditions for specific antigens. However, that study did not assess the impact of temperature during shipping. The same investigators also demonstrated that the way in which frozen PBMC are thawed, and how long PBMC are cryopreserved, will impact lymphoproliferative responses to specific antigens [26]. Bull *et al*. found that the time from phlebotomy to crypreservation should be less than 8 hours for optimal performance in cell based assays such as ELIspot and intracellular cytokine staining assays [27]. Delaying processing of whole blood by 6 hours also impaired the response of antigen-presenting cells to Toll-like receptor ligands [40]. On the other hand, Whiteside *et al. *[41] showed the phenotype and function of dendritic cell populations derived from apheresis products shipped overnight were not markedly different from DC generated from cells immediately frozen after elutriation. Smith *et al*. showed that delayed processing of blood resulted in a decrease in cell viability as well as a marked reduction in IFNγ SFC in response to varicella zoster antigen [29]; the presence of DNase partially restored the response [42]. Kierstead *et al. *[28] demonstrated that cryopreservation of PBMC should be done within 12 hours of phlebotomy. However, in these two prior studies, the whole blood [29] or PBMC [28] was stored or shipped at 4°C overnight before cryopreservation. It is not known whether there were negative effects from storing or shipping at 4°C. Our data show that there is better viability, cell yield, and function when cells are shipped at room temperature (22°C) or 30°C than at 15°C, and it is generally accepted that storage of whole blood at 4°C negatively impacts cell viability [5], function [29], and population recovery [6,7,43,44]. Acknowledging the range of data in the literature, in a separate study, we are also evaluating the function of PBMC processed the same day (< 8 h) or after overnight shipping or storage (manuscript in preparation). However the current manuscript focuses on the impact of temperature during shipping in those cases when overnight shipping is necessary.

In some prior studies, statistical differences between immediate and delayed processing of specimens were influenced not only by the delay in processing but also by the method of processing and by the type of anticoagulant used [27]. Thus, although there was a statistical decrease in viability and recovery when whole blood was collected in heparin and isolated by Accuspin technology (centrifuge tube divided into two chambers by means of a porous high-density polyethylene barrier, known as a frit), no significant decrease was evident when PBMC were collected at the interface of plasma and Ficoll. Similarly, significant differences in viability (but not recovery) between fresh and delayed samples were evident when collected in ACD or EDTA anticoagulants but not in heparin when PBMC were isolated directly onto a Ficoll cushion. Furthermore, the functionality of PBMC was not significantly impaired by either method when measured in an IFNγ-ELIspot assay in response to the CEF pool of peptides.

The observations leading to the present study come from the multi-center clinical trials we have conducted at the University of Virginia in collaboration with Cancer centers in Houston TX and Philadelphia PA. Blood specimens shipped from these locations encounter extreme seasonal climate conditions. On the other hand, blood specimens at the on-site location are, for the most part, collected, stored and processed with no exposure to extreme temperatures and processed either on the same day or after storage overnight at room temperature. This study has addressed 1) the seasonal changes in temperature inside packages of blood specimens during shipping in the U.S., 2) changes in temperature inside packages simulating hot or cold ambient temperatures during shipping, and 3) the effects of temperatures above and below room temperature on PBMC numbers, viability, and function. These studies are relevant to shipping blood specimens for correlative studies in many settings.

We are not aware of prior work tracking temperature ranges encountered within blood shipping containers or their variation by season of the year. We found that shipping of blood in insulated containers by contracted overnight carriers is associated with large seasonal variations in temperature inside the packaging, ranging from -1°C in winter to 35°C in summer, with most in the range of 4°-32°C. Thus, blood samples in transit are frequently exposed to high temperatures at or above 30°C and low temperatures that approach or go below freezing temperatures at least transiently. The monitoring devices used in these shipments recorded the minimum and maximum temperatures but not the duration of each temperature. Thus, we also studied the changes over time in a dynamic manner in hot or cold conditions designed to mimic changes that may occur during shipping, and found that insulation maintains internal temperature below 30°C for up to 21 hours in ambient temperatures that likely exceed those experienced during shipping (45°C). We found in very cold ambient conditions, that the insulated containers maintained the internal temperatures above 15°C for almost 6 hours and above 20°C for over 3 hours, with the aid of thermal packs pre-warmed at 37°C.

We have found that incubation of whole blood at 50°C caused unacceptably high loss of PBMC (data not shown). Storage at 15°C or 40°C for 12 h causes significant decreases in cell yields, viability and/or function but exposure to those temperatures for 2 hours, or in some cases even 8 hours is associated with PBMC yields, viability and function comparable to those found from blood stored at RT. The apoptosis rates in this study of about 30-35% in thawed cells incubated overnight are higher than observed in prior work where apoptosis was measured directly after thawing [32]. It is not uncommon, however, that cells undergo a delayed-onset cell death (reviewed by Baust [39]) which may account for the increase in apoptosis measured here. Other studies also confirm that the total viability decreases after overnight incubation [28]. Regardless, we find that there is function in the PBMC that are viable after overnight incubation. Incubation at 15 or 30°C is associated with comparable T cell function assessed by ELIspot assay to that seen with PBMC stored at RT. Interestingly, we found that incubation at 30°C for periods up to 12 h was even associated with equivalent or better yields, viability and function compared to samples left at RT. However, incubation at 15°C or 40°C for 8-12 h was associated with decreased viability and function. Colder temperature (15°C) primarily affected cell yield after Ficoll separation and reduced recovery following cryopreservation. Recovery may be due to a perturbation in cell density [7] or formation of cell aggregates [5,45]. No increase in apoptosis relative to that seen when blood was stored at room temperature was noted at lower temperatures which is consistent with other data showing reduced apoptotic rate of PBMC held at 4°C for 24 hours [46].

We currently endeavor to keep samples in the range of 20-30°C during shipping. In winter months, it helps to start with samples warm (e.g. 37°C) and we recommend shipping blood at 30-37°C, and recommend including a "warm pack" at 37°C in the container, which we found provides an extra 1-2 hours protection from extreme cold during winter ambient conditions (Table 3). On the other hand, during summer months, we recommend shipping at RT, to allow for some increase before exceeding 30°C. Inclusion of a fluid pack at RT may also help to buffer the temperature changes during shipping in the summer.

**Table 3:** Recommendations for shipping whole blood specimens

Time of Year	Ambient Temperature	Packing
Winter	30-37°C	37°C warm packs
Summer	RT	RT packs

We propose that controlled and monitored shipping temperatures may mitigate negative effects of shipping blood in multicenter trials. Careful attention to the shipping containers and testing in ambient temperature is recommended. Certainly one way to prevent negative effects of cold or hot temperatures during shipping of blood specimens is to isolate PBMC or other cellular elements prior to shipping, and either to cryopreserve them or to assay them on site. This introduces other sources of error and substantial costs, by the need to maintain quality control assay validation across multiple laboratories, which is problematic. We believe there is a role for shipping blood specimens for centralized assays where those assays can be performed in batches with appropriate controls, but attention to details of shipping conditions are warranted in such circumstances, to maximize the reliability of the results. It also is appropriate, in multicenter trials, to stratify patients by institution to control for systematic variations in temperature during shipping that may be encountered depending on the latitude of the institution and the shipping distance.

## Conclusions

Blood packages shipped overnight by commercial carrier may encounter extreme seasonal temperatures. Warmer temperatures favor greater cell yields of shipped blood specimens whereas colder temperatures for long periods of time lower cell recovery and viability. Temperatures ≥40C for ≥8 hours reduces cell viability and functionality after cryo-preservation. In the design of containers for blood shipment, maintaining an ambient temperature between 22°C and 30°C should be considered.

## Competing interests

JVI is a corporate entity based in Charlottesville, VA, that was contracted to make packaging for blood shipment. The CEO of JVI is Jon Fink, who is included as a co-author for his scientific contributions to package design. He is married to Robyn Fink who was a UVA employee with this research team and who managed the multicenter trials including the tracking and monitoring of blood samples shipped from outside sites. The packaging prepared by JVI to meet specifications of the research team was purchased by the University of Virginia Human Immune Therapy Center and used (when, relative to these data) for shipping blood specimens.

## Authors' contributions

WCO performed the in vitro studies, data analyses and writing the manuscript. MES performed all the statistical analysis, writing relevant sections of the manuscript and editing. EF assisted in the gathering and organization of shipping data. RJF was instrumental in concept of study. ARC assisted in the in vitro studies and data analysis. JHF designed and tested the shipping container from JVI. KAC-B developed the plan for the in vitro studies. CLS conceived study, participated in its design and coordination, and helped draft the manuscript. All authors read and approved the final manuscript.

## Supplementary Material

Additional file 1 **Comparable cell numbers were derived from complete and differential blood counts at each of the 3 hospital trial centers participating in this study**. The mean, median, 25^th ^and 75^th ^quartiles for lymphocyte and monocyte populations in the peripheral blood are presented. Values are expressed as million of cells per mL of blood. 1-Virginia; 7-Texas; 9-Pennsylvania. Click here for file

Additional file 2 **Flow diagram depicting the sequence of events in the *in Vitro *study on time and temperature of whole blood storage prior to cryopreservation and functional analysis**. Approximately 60 mL of whole blood from six healthy donors were collected into heparinized vacutainers. Aliquots were divided equally among ten conditions: nine experimental conditions in which blood was exposed to various temperatures for a defined length of time, then placed at RT (22°C) for the remainder of the 24 h storage period and one reference condition in which whole blood was stored overnight at RT. After storage, PBMC were collected after Ficoll separation, counted and cryopreserved. After 1-4 weeks, PBMC were removed for liquid nitrogen and cell recovery, viability, phenotype, and function were determined. Click here for file

Additional file 3 **Proportions of CD4 and CD8 populations in PBMC did not change under various temperature conditions, compared to RT control conditions**. Blood from 6 normal donors was incubated 24 h at RT (22°C, control), and for 2-12 h at 15, 22, 30 or 40°C (Exptl), then RT for the remainder of 24 h. PBMC were harvested, cryo-preserved, and then thawed at least 1 week later. Samples were stained with fluorescently labeled anti-CD3, anti-CD4 and anti-CD8 antibodies before flow cytometric analysis. The proportion of CD4^+ ^cells among PBMC was measured as the number of CD3^+^CD4^+ ^cells divided by the total PBMC. Similarly, the proportion of CD8+ cells among PBMC was measured as the number of CD3^+^CD8^+ ^cells divided by the total PBMC. The ratios of these CD4 and CD8 proportions are reported in this table, for each temperature condition, compared to control samples left at RT for 24 h. The estimated means, 95% confidence intervals and p-value of these ratios are shown for CD4 and CD8 populations. Click here for file

## References

[B1] Vaught JB Blood collection, shipment, processing, and storage Cancer Epidemiol Biomarkers Prev 2006 15 1582 1584 10.1158/1055-9965.EPI-06-0630 16985016

[B2] Vaught JB Caboux E Hainaut P International efforts to develop biospecimen best practices Cancer Epidemiology Biomarkers & Prevention 2010 19 912 915 10.1158/1055-9965.EPI-10-0058 20233852

[B3] Hallmans G Vaught JB Dillner J Best Practices for Establishing a Biobank Methods in Biobanking 2011 Springer Science+Business Media, LLC 241 260 full_text 10.1007/978-1-59745-423-0_13 20949394

[B4] Leyland-Jones BR Ambrosone CB Bartlett J Ellis MJC Enos RA Raji A Pins MR Zujewski JA Hewitt SM Forbes JF Abramovitz M Braga S Cardoso F Harbeck N Denkert C Jewell SD Recommendations for collection and handling of specimens from group breast cancer clinical trials J Clin Oncol 2008 26 5638 5644 10.1200/JCO.2007.15.1712 18955459 PMC2651095

[B5] Ashmore LM Shopp GM Edwards BS Lymphocyte subset analysis by flow cytometry. Comparison of three different staining techniques and effects of blood storage Journal of Immunological Methods 1989 118 209 215 10.1016/0022-1759(89)90008-2 2466904

[B6] Garraud O Moreau T Effect of blood storage on lymphocyte subpopulations Journal of Immunological Methods 1984 75 95 98 10.1016/0022-1759(84)90228-X 6512265

[B7] Weiblen BJ Debell K Giorgio A Valeri CR Monoclonal antibody testing of lymphocytes after overnight storage Journal of Immunological Methods 1984 70 179 183 10.1016/0022-1759(84)90183-2 6373939

[B8] Kaplan J Nolan D Reed A Altered lymphocyte markers and blastogenic responses associated with 24 hour delay in processing of blood samples Journal of Immunological Methods 1982 50 187 191 10.1016/0022-1759(82)90224-1 6979584

[B9] Bongers V Bertrams J The influence of common variables on T cell subset analysis by monoclonal antibodies Journal of Immunological Methods 1984 67 243 253 10.1016/0022-1759(84)90465-4 6608554

[B10] Nicholson JKA Jones BM Cross GD McDougal JS Comparison of T and B cell analyses on fresh and aged blood Journal of Immunological Methods 1984 73 29 40 10.1016/0022-1759(84)90028-0 6333461

[B11] Betensky RA Connick E Devers J Landay AL Nokta M Plaeger S Rosenblatt H Schmitz JL Valentine F Wara D Weinberg A Lederman M Shipment impairs lymphocyte proliferative responses to microbial antigens Clin Vaccine Immunol 2000 7 759 763 10.1128/CDLI.7.5.759-763.2000 PMC95951 10973450

[B12] Weinberg A Betensky RA Zhang L Ray G Effect of shipment, storage, anticoagulant, and cell separation on lymphocyte proliferation assays for human immunodeficiency virus-infected patients Clin Vaccine Immunol 1998 5 804 807 10.1128/cdli.5.6.804-807.1998 PMC96205 9801338

[B13] Lewis JJ Janetzki S Schaed S Panageas KS Wang S Williams L Meyers M Butterworth L Livingston PO Chapman PB Houghton AN Evaluation of CD8+ T-cell frequencies by the Elispot assay in healthy individuals and in patients with metastatic melanoma immunized with tyrosinase peptide Int J Cancer 2000 87 391 398 10.1002/1097-0215(20000801)87:3<391::AID-IJC13>3.0.CO;2-K 10897045

[B14] Scheibenbogen C Lee K-H Stevanovic S Witzens M Waldmann V Naeher H Rammensee H-G Keilholz U Analysis of the T cell response to tumor and viral peptide antigens by an IFN[gamma]-elispot assay Int J Cancer 1997 71 932 936 10.1002/(SICI)1097-0215(19970611)71:6<932::AID-IJC3>3.0.CO;2-Z 9185691

[B15] Schmittel A Keilholz U Scheibenbogen C Evaluation of the interferon-[gamma] ELISPOT-assay for quantification of peptide specific T lymphocytes from peripheral blood Journal of Immunological Methods 1997 210 167 174 10.1016/S0022-1759(97)00184-1 9520299

[B16] Jung T Schauer U Heusser C Neumann C Rieger C Detection of intracellular cytokines by flow cytometry Journal of Immunological Methods 1993 159 197 207 10.1016/0022-1759(93)90158-4 8445253

[B17] Letsch A Scheibenbogen C Quantification and characterization of specific T-cells by antigen-specific cytokine production using ELISPOT assay or intracellular cytokine staining Methods 2003 31 143 149 10.1016/S1046-2023(03)00124-5 12957572

[B18] Maecker HT Dunn HS Suni MA Khatamzas E Pitcher CJ Bunde T Persaud N Trigona W Fu TM Sinclair E Bredt BM McCune JM Maino VC Kern F Picker LJ Use of overlapping peptide mixtures as antigens for cytokine flow cytometry Journal of Immunological Methods 2001 255 27 40 10.1016/S0022-1759(01)00416-1 11470284

[B19] Maino VC Maecker HT Cytokine flow cytometry: a multiparametric approach for assessing cellular immune responses to viral antigens Clinical Immunology 2004 110 222 231 10.1016/j.clim.2003.11.018 15047200

[B20] Britten C Janetzki S Ben-Porat L Clay T Kalos M Maecker H Odunsi K Pride M Old L Hoos A Romero P for the HLA-peptide Multimer Proficiency Panel of the CVC-CRI Immune Assay Working Group Harmonization guidelines for HLA-peptide multimer assays derived from results of a large scale international proficiency panel of the Cancer Vaccine Consortium Cancer Immunology, Immunotherapy 2009 58 1701 1713 10.1007/s00262-009-0681-z PMC2714899 19259668

[B21] Frelinger J Ottinger J Gouttefangeas C Chan C Modeling flow cytometry data for cancer vaccine immune monitoring Cancer Immunology, Immunotherapy 2010 59 1435 1441 10.1007/s00262-010-0883-4 PMC2892609 20563720

[B22] Altman JD Moss PAH Goulder PJR Barouch DH Heyzer-Williams MG Bell JI McMichael AJ Davis MM Phenotypic analysis of antigen-specific T lymphocytes Science 1996 274 94 96 10.1126/science.274.5284.94 8810254

[B23] Axelsson S Faresj÷ M Hedman M Ludvigsson J Casas R Cryopreserved peripheral blood mononuclear cells are suitable for the assessment of immunological markers in type 1 diabetic children Cryobiology 2008 57 201 208 10.1016/j.cryobiol.2008.08.001 18761006

[B24] Kreher CR Dittrich MT Guerkov R Boehm BO Tary-Lehmann M CD4+ and CD8+ cells in cryopreserved human PBMC maintain full functionality in cytokine ELISPOT assays Journal of Immunological Methods 2003 278 79 93 10.1016/S0022-1759(03)00226-6 12957398

[B25] Maecker H Moon J Bhatia S Ghanekar S Maino V Payne J Kuus-Reichel K Chang J Summers A Clay T Morse M Lyerly HK DeLaRosa C Ankerst D Disis M Impact of cryopreservation on tetramer, cytokine flow cytometry, and ELISPOT BMC Immunology 2005 6 1 17 10.1186/1471-2172-6-1 16026627 PMC1190174

[B26] Weinberg A Song LY Wilkening C Sevin A Blais B Louzao R Stein D Defechereux P Durand D Riedel E Raftery N Jesser R Brown B Keller MF Dickover R McFarland E Fenton T for the Pediatric ACTG Cryopreservation Working Group Optimization and limitations of use of cryopreserved peripheral blood mononuclear cells for functional and phenotypic T-cell characterization Clin Vaccine Immunol 2009 16 1176 1186 10.1128/CVI.00342-08 19515870 PMC2725535

[B27] Bull M Lee D Stucky J Chiu YL Rubin A Horton H McElrath MJ Defining blood processing parameters for optimal detection of cryopreserved antigen-specific responses for HIV vaccine trials Journal of Immunological Methods 2007 322 57 69 10.1016/j.jim.2007.02.003 17382342 PMC1904432

[B28] Kierstead LS Dubey S Meyer B Tobery TW Mogg R Fernandez VR Long R Guan L Gaunt C Collins K Sykes KJ Mehrotra DV Chirmule N Shiver JW Casimiro DR Enhanced rates and magnitude of immune responses detected against an HIV vaccine: Effect of using an optimized process for isolating PBMC AIDS Research and Human Retroviruses 2007 23 86 92 10.1089/aid.2006.0129 17263637

[B29] Smith JG Levin M Vessey R Chan ISF Hayward AR Liu X Kaufhold RM Clair J Chalikonda I Chan C Bernard M Wang WW Keller P Caulfield MJ Measurement of cell-mediated immunity with a varicella-zoster virus-specific interferon-+¦ ELISPOT assay: Responses in an elderly population receiving a booster immunization J Med Virol 2003 70 S38 S41 10.1002/jmv.10318 12627485

[B30] Disis ML dela Rosa C Goodell V Kuan LY Chang JCC Kuus-Reichel K Clay TM Kim Lyerly H Bhatia S Ghanekar SA Maino VC Maecker HT Maximizing the retention of antigen specific lymphocyte function after cryopreservation Journal of Immunological Methods 2006 308 13 18 10.1016/j.jim.2005.09.011 16337957

[B31] Weinberg A Song LY Wilkening CL Fenton T Hural J Louzao R Ferrari G Etter PE Berrong M Canniff JD Carter D Defawe OD Garcia A Garrelts TL Gelman R Lambrecht LK Pahwa S Pilakka-Kanthikeel S Shugarts DL Tustin NB Optimization of storage and shipment of cryopreserved peripheral blood mononuclear cells from HIV-infected and uninfected individuals for ELISPOT assays Journal of Immunological Methods 2010 363 42 50 10.1016/j.jim.2010.09.032 20888337 PMC3068047

[B32] Smith JG Joseph HR Green T Field JA Wooters M Kaufhold RM Antonello J Caulfield MJ Establishing acceptance criteria for cell-mediated-immunity assays using frozen peripheral blood mononuclear cells stored under optimal and suboptimal conditions Clin Vaccine Immunol 2007 14 527 537 10.1128/CVI.00435-06 17376862 PMC1865640

[B33] Slingluff CL Petroni GR Chianese-Bullock KA Smolkin ME Hibbitts S Murphy C Johansen N Grosh WW Yamshchikov GV Neese PY Patterson JW Fink R Rehm PK Immunologic and clinical outcomes of a randomized phase II trial of two multipeptide vaccines for melanoma in the adjuvant setting Clin Cancer Res 2007 13 6386 6395 10.1158/1078-0432.CCR-07-0486 17975151

[B34] Slingluff CL Petroni GR Olson WC Smolkin ME Ross MI Haas NB Grosh WW Boisvert ME Kirkwood JM Chianese-Bullock KA Effect of granulocyte/macrophage colony-stimulating factor on circulating CD8+ and CD4+ T-cell responses to a multipeptide melanoma vaccine: outcome of a multicenter randomized trial Clin Cancer Res 2009 15 7036 7044 10.1158/1078-0432.CCR-09-1544 19903780 PMC2778314

[B35] Currier JR Kuta EG Turk E Earhart LB Loomis-Price L Janetzki S Ferrari G Birx DL Cox JH A panel of MHC class I restricted viral peptides for use as a quality control for vaccine trial ELISPOT assays Journal of Immunological Methods 2002 260 157 172 10.1016/S0022-1759(01)00535-X 11792386

[B36] Martin SJ Reutelingsperger CPM McGahon AM Rader JA van Schie RC LaFace DM Green DR Early redistribution of plasma membrane phosphatidylserine is a general feature of apoptosis regardless of the initiating stimulus: inhibition by overexpression of Bcl-2 and Abl Journal of Experimental Medicine 1995 182 1545 1556 10.1084/jem.182.5.1545 7595224 PMC2192182

[B37] Schmid I Uittenbogaart CH Keld B Giorgi JV A rapid method for measuring apoptosis and dual-color immunofluorescence by single laser flow cytometry Journal of Immunological Methods 1994 170 145 157 10.1016/0022-1759(94)90390-5 8157993

[B38] Vermes I Hannen C Steffens-Nakken H A novel assay for apoptosis. Flow cytometric detection of phosphatigylserine expression on early apoptotic cells using fluorescein labelled Annexin V Journal of Immunological Methods 1995 184 39 51 10.1016/0022-1759(95)00072-I 7622868

[B39] Baust JM Molecular mechanisms of cellular demise associated with cryopreservation failure Cell Preservation Technology 2002 1 17 31 10.1089/15383440260073266

[B40] Meier A Fisher A Sidhu HK Chang JJ Wen TF Streeck H Alter G Silvestri G Altfeld M Rapid loss of dendritic cell and monocyte responses to TLR ligands following venipuncture J Immunol Methods 2008 339 132 140 10.1016/j.jim.2008.09.007 18848564 PMC2613681

[B41] Whiteside TL Griffin DL Stanson J Gooding W McKenna D Sumstad D Kadidlo D Gee A Durett A Lindblad R Wood D Styers D Shipping of therapeutic somatic cell products Cytotherapy 2010 1 13 Early Online 10.3109/14653249.2010.506507 PMC7982143 20795760

[B42] Smith JG Liu X Kaufhold RM Clair J Caulfield MJ Development and validation of a gamma interferon ELISPOT assay for quantitation of cellular immune responses to varicella-zoster virus Clin Vaccine Immunol 2001 8 871 879 10.1128/CDLI.8.5.871-879.2001 PMC96163 11527795

[B43] McKenna KC Beatty KM Vicetti Miguel R Bilonick RA Delayed processing of blood increases the frequency of activated CD11b+ CD15+ granulocytes which inhibit T cell function Journal of Immunological Methods 2009 341 68 75 10.1016/j.jim.2008.10.019 19041316

[B44] Van Lambalgen R Van Meurs GJE Lymphocyte subpopulations do not alter during blood storage at 4C Journal of Immunological Methods 1985 80 39 43 10.1016/0022-1759(85)90162-0 3159799

[B45] De Paoli P Villalta D Battistin S Gasparollo A Santini G Letter to the editor Journal of Immunological Methods 1983 61 259 260 10.1016/0022-1759(83)90170-9 6602852

[B46] Bergman M Bessler H Salman H Djaldetti M Relationship between temperature and apoptosis of human peripheral blood mononuclear cells International Journal of Hematology 2003 77 351 353 10.1007/BF02982642 12774922

