# Supplementary material for: Shipping blood to a central laboratory in multicenter clinical trials: effect of ambient temperature on specimen temperature, and effects of temperature on mononuclear cell yield, viability and immunologic function
Source: J Transl Med. 2011 Mar 8;9:26. doi: 10.1186/1479-5876-9-26 (PMC3063218; doi:10.1186/1479-5876-9-26)
Supplement: Additional file 1 — Comparable cell numbers were derived from complete and differential blood counts at each of the 3 hospital trial centers participating in this study. The mean, median, 25th and 75th quartiles for lymphocyte and monocyte populations in the peripheral blood are presented. Values are expressed as million of cells per mL of blood. 1-Virginia; 7-Texas; 9-Pennsylvania. [file 1479-5876-9-26-S1.PDF]

	Lymphocytes					Monocytes				
**Hospital**	**N**	**Mean**	**Median**	**25%**	**75%**	**N**	**Mean**	**Median**	**25%**	**75%**
**1**	212	1.65	1.55	1.22	2.11	217	0.53	0.47	0.36	0.63
**7**	232	1.56	1.52	1.20	1.89	232	0.48	0.49	0.38	0.58
**9**	81	1.65	1.70	1.34	2.00	81	0.50	0.50	0.40	0.60
**Total**	525	1.61	1.56	1.22	1.98	530	0.51	0.48	0.37	0.59
