# Supplementary material for: Shipping blood to a central laboratory in multicenter clinical trials: effect of ambient temperature on specimen temperature, and effects of temperature on mononuclear cell yield, viability and immunologic function
Source: J Transl Med. 2011 Mar 8;9:26. doi: 10.1186/1479-5876-9-26 (PMC3063218; doi:10.1186/1479-5876-9-26)
Supplement: Additional file 2 — Flow diagram depicting the sequence of events in the in Vitro study on time and temperature of whole blood storage prior to cryopreservation and functional analysis. Approximately 60 mL of whole blood from six healthy donors were collected into heparinized vacutainers. Aliquots were divided equally among ten conditions: nine experimental conditions in which blood was exposed to various temperatures for a defined length of time, then placed at RT (22°C) for the remainder of the 24 h storage period and one reference condition in which whole blood was stored overnight at RT. After storage, PBMC were collected after Ficoll separation, counted and cryopreserved. After 1-4 weeks, PBMC were removed for liquid nitrogen and cell recovery, viability, phenotype, and function were determined. [file 1479-5876-9-26-S2.PDF]

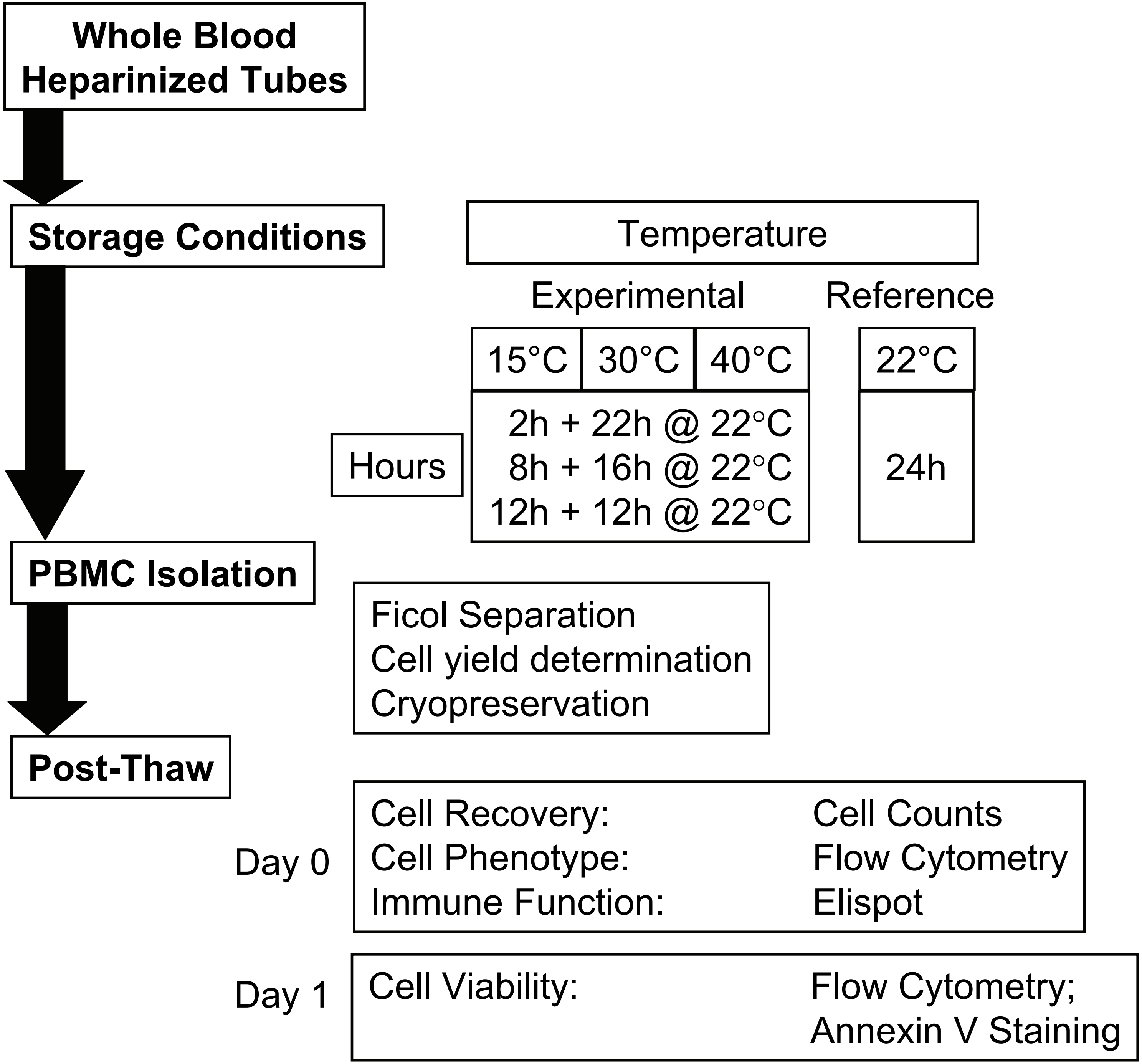
Whole Blood
Heparinized Tubes
Storage Conditions
Temperature
Experimental
Reference
15°C
30°C
40°C
22°C
Hours
2h + 22h @ 22°C
8h + 16h @ 22°C
12h + 12h @ 22°C
24h
PBMC Isolation
Ficol Separation
Cell yield determination
Cryopreservation
Post-Thaw
Day 0
Cell Recovery:
Cell Phenotype:
Immune Function:
Cell Counts
Flow Cytometry
Elispot
Day 1
Cell Viability:
Flow Cytometry;
Annexin V Staining
