# Supplementary material for: Shipping blood to a central laboratory in multicenter clinical trials: effect of ambient temperature on specimen temperature, and effects of temperature on mononuclear cell yield, viability and immunologic function
Source: J Transl Med. 2011 Mar 8;9:26. doi: 10.1186/1479-5876-9-26 (PMC3063218; doi:10.1186/1479-5876-9-26)
Supplement: Additional file 3 — Proportions of CD4 and CD8 populations in PBMC did not change under various temperature conditions, compared to RT control conditions. Blood from 6 normal donors was incubated 24 h at RT (22°C, control), and for 2-12 h at 15, 22, 30 or 40°C (Exptl), then RT for the remainder of 24 h. PBMC were harvested, cryo-preserved, and then thawed at least 1 week later. Samples were stained with fluorescently labeled anti-CD3, anti-CD4 and anti-CD8 antibodies before flow cytometric analysis. The proportion of CD4+ cells among PBMC was measured as the number of CD3+CD4+ cells divided by the total PBMC. Similarly, the proportion of CD8+ cells among PBMC was measured as the number of CD3+CD8+ cells divided by the total PBMC. The ratios of these CD4 and CD8 proportions are reported in this table, for each temperature condition, compared to control samples left at RT for 24 h. The estimated means, 95% confidence intervals and p-value of these ratios are shown for CD4 and CD8 populations. [file 1479-5876-9-26-S3.PDF]

CD4 Ratio (Compared to RT Control)			
**Exptl**	**2h**	**8h**	**12h**
**RT**	**22h**	**16h**	**12h**
**15°C**	1.03<br>(0.93, 1.13)<br>p=0.51	1.05<br>(0.95, 1.15)<br>p=0.28	1.05<br>(0.95, 1.15)<br>p=0.27
**30°C**	1.03<br>(0.93, 1.13)<br>p=0.54	0.95<br>(0.85, 1.05)<br>p=0.28	0.97<br>(0.88, 1.07)<br>p=0.57
**40°C**	1.00<br>(0.90, 1.10)<br>p=0.94	1.00<br>(0.90, 1.10)<br>p=0.98	1.03<br>(0.93, 1.13)<br>p=0.53

CD8 Ratio (Compared to RT Control)			
**Expt**	**2h**	**8h**	**12h**
**RT**	**22h**	**16h**	**12h**
**15°C**	0.95<br>0.86, 1.04)<br>p=0.27	0.93<br>(0.84, 1.02)<br>p=0.11	0.96<br>(0.87, 0.1.04)<br>p=0.29
**30°C**	0.98<br>(0.89, 1.07)<br>p=0.62	1.00<br>(0.91, 1.09)<br>p=0.95	1.01<br>(0.92, 1.10)<br>p=0.84
**40°C**	0.99<br>(0.90, 1.07)<br>p=0.73	0.96<br>(0.87, 1.05)<br>p=0.34	0.96<br>(0.86, 1.05)<br>p=0.33
